# “She’s Always Made Sure That We Had Black Doctors, Particularly Women Doctors If We Could… and How It Can Sometimes Be the Difference Between Life and Death.” Black College Women’s Reflections On Medical Racism As a Social Determinant of Health

**DOI:** 10.1007/s40615-025-02366-0

**Published:** 2025-03-07

**Authors:** Jeannette Marie Wade, Helyne Frederick, Sharon Parker

**Affiliations:** 1 Human Health Sciences, University of North Carolina-Greensboro, Greensboro, USA; 2 Human Development and Family Science, University of North Carolina at Chapel Hill, Chapel Hill, USA; 3 North Carolina a&T State University, Greensboro, USA

**Keywords:** Medical racism, Vicarious racism, Black women’s health, Sexual and reproductive health

## Abstract

Historical instances of medical racism and the impact of ongoing disparities are an understudied determinant of Black women’s sexual health. Here, we use a Black feminist approach to engage Black women in a qualitative exploration of the impact of medical racism on their health-related decision-making. Specifically, we explore the question, how does exposure to information on medical racism impact Black women today and inform their perceptions of healthcare? This qualitative study uses Black feminist approaches to study design including advisory boards, interviewer concordance, and focus group data collection with Black female college students who were in the developmental phase of emerging adulthood, ages 18–25. We also led seven virtual focus groups that focused on dialogue around agents of sexual socialization including knowledge of historical and ongoing medical racism. Four themes emerged from our thematic analysis. The first theme was compromised mental health. The second theme was a wealth and health paradox whereby income and education did not mitigate the impact of racism on health. The third theme was medical racism and distrust. And the fourth theme was around the role of Black Americans as guinea pigs, autonomous actors, and advocates in healthcare. Medical racism, whether it is experienced firsthand, vicariously, or as a part of one’s history, is a source of anxiety for Black women. This barrier to care must be addressed to promote health equity in the USA.

## Introduction

Black women in America face stark sexual and reproductive health disparities compared to other racial groups [1]. For instance, the Centers for Disease Control and Prevention (CDC) reported that chlamydia rates among Black female adolescents are six times higher than those among their White female counterparts, and that this disparity extends into young adulthood as well [2]. Similarly, Black women are 30% more likely to develop, and 60% more likely to die from, cervical cancer when compared to non-Hispanic White women [3]. Additionally, they are overrepresented among women who seek prenatal care late, or never (at 10% compared to 5% of white women), have higher rates of unintended pregnancies (at 79/1000 live births compared to 33/1000 among white women), and have higher maternal mortality rates (at 41/100 k live births compared to 13/100 k live births among white women) [4]. Trends in Black women’s sexual health can be attributed to numerous factors including the social determinants of health (SDOH). The CDC describes the SDOH as non-medical factors that influence health, which are often manifestations of racism. Examples of SDOH include neighborhood-level constraints like access to food, pharmacies, properly funded schools, jobs with livable wages, and transportation [5].

We define racism as a system where oppression and opportunity are garnered based on skin color, hair texture, and historical caste systems that placed descendants of Europe at the top and descendants of Africa at the bottom. Racism itself, however, is also noted as a key determinant of health [6]. In fact, in a meta-analysis of over 300 studies, exposure to racism was shown to impact both mental and physical health across all race groups [7]. Given the persistent nature of both Black women’s sexual health disparities, it is vital to gain qualitative insights into Black women’s perceptions of racism and its impact on their health-related decision-making. Qualitative data collected during focus groups allows Black women to share their lived experiences in an open forum that is not constrained to limited options as is the case with survey methods. Here, we use a Black feminist approach to consider the impact of having information on medical racism and the intergenerational transfer of historical racism on Black women’s sexual health and attitudes around the system of healthcare in America. Black feminist thought calls for research that (1) centers Black women, (2) accounts for socio-cultural and social psychological factors that shape Black women’s experiences, and (3) utilizes Black female scholars who have shared lived experiences with their participants and are better suited to ensure qualitative findings are properly interpreted, and not presented in a deficit focused manner [8, 9].

### Racism and Health

In terms of the system of healthcare, racism, sometimes referred to as medical racism, manifests in several ways. It can be systemic, as described by the SDOH, where access to resources with known connections to health is limited for some and abundant for others. It can be interpersonal, where patient-provider interactions are based on racial differences and driven by stereotypes [10–12]. Racism in healthcare can be gender specific, where stereotypes about sex, gender, and race impact patient care simultaneously [13, 14]. For example, Black women have been socially constructed as oversexed, jezebels [15], and the Black women interviewed by Wade et al. [12] discussed how gynecologists would assume they were oversexed and at elevated risk for pregnancy without securing any details about them.

Medical racism can also be historical and vicarious. Historical racism is particularly relevant to studies of Black health as Black Americans have a unique and sordid history with healthcare and medical research [16]. According to Gee and Ford [17], historical trauma can have an intergenerational impact on population health. In the case of Black women, examples of such trauma include the Alabama Fistula Study, where slave women were forced into vaginal surgical trials prior to the invention of anesthesia, and the Tuskegee Study of Untreated Syphilis in the Negro Male, where the life course of syphilis was examined, in a sample of uninformed Black men, which resulted in the infection of two generations of women and children, and for North Carolinians, in particular, attitudes towards gynecological care were shaped by stories of over 7600 poor Black women being forcibly sterilized [18, 19].

As a result of the multifaceted nature of medical racism, Black Americans have a heightened sense of medical distrust [20, 21]. Medical distrust leaves Black Americans more likely to (1) avoid sexual healthcare providers as well as (2) engage in self-preservation acts like postponing pregnancy—to delay time spent in medical facilities [22]. There has been much work attributing Black health outcomes of today, and related mistrust to historical events like those listed above [18]. However, other studies are emerging that call previous suppositions into question. Brandon, Isaac, and LaVeist [23], for example, found quantitative support against the notion that Black medical mistrust is connected to the events in Tuskegee. They allude to a potentially greater impact of vicarious racism, over historical racism. Vicarious racism is conceptualized as indirect exposure to racism as experienced by close loved ones or distal racemates (as in strangers whose stories are made public), which can have a distressing impact on the person who experiences it [24]. Although historical racism can be considered vicarious, the key difference is historical time. The question becomes are Black women’s views of health and healthcare impacted by historical racism of the distant past (like the experiences of enslaved women), vicarious racism of the present (like stories from loved ones or social media), or both to some degree, and how do interventionists and medical professionals mitigate and address those impacts?

### Theory: Black Feminist Approach

The central tenet of Black Feminist thought is that Black women are a unique group of Americans as members of two marginalized populations. According to Collins [9], this means that as Black Americans, the racism of the past and present impacts Black women’s lived experiences. And that as women, gender oppression of the past and present impacts Black women’s lived experiences. And as Black women in America, acts of gendered racism, or misogynoir, of the past and present impact Black women’s lived experiences. Brantley’s [25] conceptual framework on Black feminist approaches to maternal health research offers much perspective to studies of sexual healthcare as well. Specifically, she suggested scholars (1) center Black women, by including them on research teams and citing Black female scholars; (2) acknowledge the ways in which health is linked to systems of oppression, here racism, both historical and vicarious; and (3) use Black feminist theory to make connections between the history of Black womanhood and how it impacts Black womanhood today. Here, we use this Black feminist epistemology and methodology to engage Black women in a qualitative exploration of Black women’s knowledge of and experiences with medical racism influence the healthcare-related anxiety they experience. Specifically, we explore the question, how does knowledge of historical medical racism impact Black women today?

## Methods

The current study used data from a larger project, “An Exploratory Study of Barriers to Black Women’s Involvement in Gynecological Research and Healthcare” conducted at a Historically Black College and University (HBCU) and a Predominantly White Institution (PWI) in the southeastern region of the USA. Both campuses provided IRB approval for the study in 2019. The sample was limited to Black female college students between the ages of 18 and 25, who identified as heterosexual. Those inclusion criteria are in place to (1) understand the Black female experience during emerging adulthood and (2) focus on gendered racism without potentially conflating homophobia. Although Black women’s perceptions of historical medical racism may not vary based on sexual orientation, the larger study included explorations of discrimination in healthcare and related research which is where homophobia could potentially be conflated. Emerging adulthood, ages 18–25, is an important developmental phase to focus on as young Americans are most likely to engage in high-risk sexual behaviors as they transition from adolescents to adults [26]. It is also important to focus on a particular cohort of women to see how history has impacted their experience with sexual socialization and perceptions of healthcare.

We employed Black feminist methodology by Collins [9] and Lindsay-Dennis [27] as a guiding framework for the study. Lindsay-Dennis [27] called for research that considers literature outside one’s own discipline to understand the multifaceted nature of Black womanhood. Here, we engage literature from sociology, history, family studies, and psychology to do just that. Collins [9] noted that “Black women intellectuals are central to Black feminist thought for several reasons one of which is their ability to empathize with and relate to psychosocial aspects of Black womanhood in ways that ‘those who live outside those structures cannot’” (p. 35). Additionally, a recent study by Frederick et al. [8] demonstrated that the engagement of young Black women in sexual healthcare research significantly improves when approached by trusted community members, particularly Black female researchers. As such, we used a Black female research team to recruit and engage our participants. The team consisted of three faculty investigators, a research assistant and two volunteer undergraduate assistants. Lindsay-Dennis also adds that shared demographics is not enough to engage Black female participants [27]. One must also display a level of concern and an ethic of care. This can be more challenging for PWIs whose history includes segregation and manipulation of research subjects [18]. In developing a connection and building trust, the team had to pivot from the use of stock images to real photos of the research team on marketing materials. This helped build trust at the PWI as potential participants were concerned about the races of the researchers. Therefore, we believe that our strategies embodied the Black feminist framework and allowed us to center the experiences of Black college women. We used the campus listservs and social media to recruit the participants for the study. In preparation for the study, we engaged a Black female student advisory board to ensure the study was culturally tailored, gender specific, and relevant to college students. The board consisted of five 18–25-year-old Black women, from both campuses, who were undergraduate and graduate students. Student board members were active in campus organizations and thus able to assist with recruiting and retaining participants, pilot testing study protocols, and ensuring our analyses were, empowering, and not deficit focused which aligns with Black feminist practices.

### Data Collection

We conducted seven focus groups with the women from the HBCU (*n* = 20) and PWI (*n* = 8). We interviewed the women from both institutions together. The focus groups provided Black women the opportunity to share their perspectives on the sexual socialization they received as well as their knowledge and reflection on historical trauma. All focus groups were conducted over a video platform. Each focus group included two lead PIs and a student research assistant. All women consented to be video/audio recorded. We told women that the information was sensitive and that they could stop the focus group at any time. We also ensured all participants that their responses would be de-identified and asked the women to refrain from sharing details of the focus group outside of the focus group setting. They were also made aware of resources and support from the campus counseling services. We engaged in several warm-up activities to build rapport and establish connections with the research teams and other focus group participants. One of the team members provided an overview of Black history in medicine to ensure each participant could thoughtfully engage in a discussion of the impact of historical racism. The carefully curated presentation covered the forced sterilization of Black women, Dr. Marion Sims and the C-section trials, the U.S. Public Health Service (USPHS) Untreated Syphilis Study at Tuskegee, as well as recent data related to Black women’s sexual and reproductive health. Some participants learned about these events for the first time, while others received a refresher. At the conclusion of the presentation, we asked women to reflect on the material. They were encouraged to discuss how these historical events resonated with their personal experiences, how they view the healthcare system today, and how medical racism impacted their interactions with providers. It is worth noting that although the PowerPoint presentation was used as an educational tool, it may have also had unintended priming effects. Specifically, women, who (1) were unfamiliar with the history of medical racism or (2) had not spent time processing the events in the context of their own health, may have experienced increased anxiety and fears of future harm at the hands of healthcare providers. We believe that educating the group was in the best interest of all and a great way to promote dialogue among the focus group participants. However, we also acknowledge that this can be considered a limitation to the study and believe future work is needed that assess the impact of history—as they know it, not as they learn about it in real time during a session.

## Data Analysis

We used a paid transcription service provider, to transcribe the focus groups. The research team listened to recordings and reviewed the transcripts for accuracy. All audio files were transcribed and de-identified to ensure accuracy and anonymity. Our analytical approach focused on Black Feminist perspectives as well as inductive thematic analysis. In line with Black feminist inquiry, we included Black female research assistants in the analysis process who were also emerging adults like our respondents. This helped bridge a potential age between the PIs and the respondents to ensure responses were not misrepresented. We also collectively processed our own traumas that arose during analysis as a team of Black women who have also been impacted by medical racism. Each of the three PIs and two student assistants worked to code the focus groups. Braun and Clark [28] outlined six steps to conducting inductive thematic analysis and analyzing the narratives provided by the participants. Our first step involved familiarizing ourselves with the data by reading the manuscripts and taking notes. We also considered the memos taken during the interview process. In the second step, the three investigators independently reviewed the narratives and generated codes using a matrix that documented codes with sample codes from each of the transcripts. In step 3, the investigators reviewed the matrix to examine commonalities with the codes and used these codes to generate initial themes. What followed next was a thorough review of the themes and codes to ensure that the data and the themes aligned. The process involved combining themes and resolving discrepancies or concerns with the data (steps 4 and 5). The sixth step involved writing the themes for manuscript preparation. Our incorporation of Bruan and Clark’s [28] 15-step checklist is included in Appendix 1.

### Trustworthiness and Reflexivity

As Black female investigators researching a sensitive topic about Black women, we reflected on our positionality and the potential for bias. Lincoln and Guba [29] encourage qualitative investigators to prioritize trustworthiness and establish credibility with the research process. We took several steps to minimize bias and bolster rigor in the research process. We engaged in memo writing and frequent meetings to discuss our process and findings. We collaborated with our advisory board of Black women college students to generate the questions used in the study. During the entire process, we discussed our own positionalities and opinions about the focus group process and followed the framework of Black feminist scholarship in our work.

## Results

Women’s familiarity with data about sexual and gynecological health, current and past, varied. The women majoring in the social sciences tended to be familiar with some of the issues presented. Most of them did not study the issues in depth and were appreciative of the contextual information provided in the focus group. We derived four major themes from the analysis of the focus group data. The themes were compromised mental health, wealth and health paradox, medical distrust and racism, and the role of Black Americans. These themes reflected the Black women’s concerns about inferior quality healthcare and interaction with providers. The themes also highlighted how past traumatic events and current realities of Black women’s negative maternal and other health concerns may be contributing to the anxiety that Black women experience regarding healthcare.

### Compromised Mental Health

The first theme from our analysis was compromised mental health. We conceptualize compromised mental health as references to distress, anxiety, anger, fear, or any feeling that leads to poor mental health and/or physiological responses. After the women were presented with known mistreatment of Black women in sexual healthcare, they were asked to respond to what they saw and heard. All the women noted that even though several events happened many years ago, they felt distressed and uncomfortable knowing that race impacted how Black women were treated. The more recent data they saw on current disparities left them feeling distressed as Black women of childbearing age. Our participants also noted that despite the information being difficult to hear and process, they were relieved to know that educators are discussing these issues at least in the college classroom.

History teachers were becoming more comfortable with talking about how Black people were really treated instead of just following the actual curriculum guideline. These events are unsettling to me. - Lisa

I think that those experiments it’s very heavy. It’s definitely a weight that we have to carry with us. - Kesha

Here, we see two examples of young Black women who are experiencing poor psychological and physiological sensations that stem from exposure to instances of historical and vicarious racism. A Black feminist analysis of theme one would call for an interrogation of the system of healthcare which is rooted in and structured by white dominance and patriarchy. Collins [8] coined the term controlling images to connote the racist, sexist stereotypes that plague Black women and shape the way they experience social structures like medicine. Here, Black women are seen as too strong to experience pain, too savage to deserve ethical treatment in clinical trials, and too oversexed to experience concern and quality patient-provider interactions. One cost of this treatment is intergenerational trauma and compromised mental health. Here, we see even vicarious or historical instances of racism leading to distress in the same ways firsthand experiences would.

### Wealth and Health Paradox

The second theme from our analysis was the wealth and health paradox. The wealth and health paradox describes the realities faced by Black women in the USA. We operationalize the wealth and health paradox as health outcomes that are counter to existing frameworks around the relationships between health and social class. The Fundamental Cause Theory is an example that posits that social conditions are the greatest predictors of health; thus, the more educated and financially stable individuals are more likely they are to receive quality healthcare and have better health [30]. In medical sociological literature, there is a term “countervailing mechanism” that came from a study of wealthy, White women who chose not to take their needed medication because it came with the potential risk of weight gain—and they valued having a thin frame over being healthy. A countervailing mechanism is an outside force or social driver that takes priority over health, even when individuals have access to quality healthcare [31]. Here, we use the term paradox because Black women with wealth experience poor care due to racism, not a competing aspect of status or by choice.

The notion that being financially well-off should equate to having positive experiences in healthcare was discussed. The women acknowledged that even famous and popular Black women, like Serena Williams and Beyonce, experienced challenges with pregnancy and that if these women had challenges, then they were likely to as well. Our participants were aware that being educated did not shield them from having challenges with healthcare and poor maternal health outcomes.

I remember hearing about Serena Williams and Beyonce, and how they have their pregnancy complications, and that terrified me because I’m just like, these women are the richest Black women ever and they’re having pregnancy complications. What does that mean for me? That terrified me to have kids. - Nia

I’m just like, I thought we were trying to get more college-educated, we’re trying to wait longer, and be more informed so that we can reduce these risks. But it was like, the stress from racism that occurs in the workforce is aging us and the more college-educated we are, we’re more at risk to have complications. - Erica

The health and wealth paradox is another example of a cost of the controlling images in place to marginalize Black women. The notion that Black women are strong, and do not experience pain at the same rates as white women, is so pervasive that not even fame or fortune can mitigate its impact.

### Medical Distrust and Racism

The third theme that emerged from our analysis was medical distrust and racism. The sense of distrust seemed to come from the socialization Black women received within their own families. They discussed how family members steered them towards finding Black healthcare professionals after having poor experiences with non-Black healthcare providers. Several participants were already aware that race intertwined with healthcare experiences and that these experiences were part of Black history and persist in the present day. They were keenly aware of the role that Black women played in advancing medical knowledge and the expense of their health and well-being. Here, we see two manifestations of vicarious racism. In one case, the respondent took the words of her mom and used them as motivation to seek out Black doctors for all her healthcare needs. In the other case, a participant took her aunt’s experience and used it to contextualize existing disparities.

#### Medical Distrust

Growing up, like, my mom always made sure every doctor I’ve ever had in my life was Black. From my dentist to my dermatologist, she would always just explain to us why she’s always made sure that we had Black doctors, particularly women doctors if we could… and how it can sometimes be the difference between life and death. - Gina

#### Racism

I could definitely see there’s definitely a disparity when it comes to Black women and white women within the healthcare industry, just based on how my aunt was treated. She had to change the hospital and her doctors quite often, just because she was getting older white men who were telling her things, and it was just... they were combating each other in a way. -Lisa

Medical distrust and medical racism are health-related manifestations of larger systems in place to perpetuate white male supremacy. A Black feminist perspective of medical distrust would connect Black women’s skepticism to historical events like those highlighted in the focus groups, as well as the experiences of Black women study participants themselves, and their loved ones [9]. Black feminist scholars would call for dismantling the current system of care and establishing one where Black women’s experiences are centered and perceived as viable actors navigating their own health journey.

### Role of Black Americans

The fourth theme, the role of Black Americans, showed the diverse ways Black women conceptualized the role of Black Americans in terms of health and healthcare. The subthemes included medical guinea pigs, empowered, and autonomous actors. First, several participants expressed frustrations around the misuse of Black Americans in health-related research; for example, Kimi stated, “Black people have been at the hands of white nonsense for centuries and it’s just… it’s disgusting.”

Second, from a vastly different perspective, several participants saw historical and ongoing medical racism as a reason to be empowered and advocate for others. The empowered subtheme demonstrates Black women’s sense of agency and purpose. They also noted that it was important for them to be advocates and stand stronger for other Black women. For these women, having the knowledge and awareness empowered them to take the necessary steps towards improving their own health experiences and outcomes.

It also makes me want to also stand stronger for women and stand up for Black women, us as women and our sexuality and our identities. - Raye

Third, several Black women saw this as a call to take charge of their own health or be autonomous health actors. They indicated that knowing about historical and present traumas in healthcare means that they must take the initiative on preventative care. Specifically, Chell noted, “because in the Black community parents say, go get tested, but it’s not like the thing where everyone is protesting. People don’t actually go as often as they should…We have to promote it more to where we make it as testing isn’t a bad thing. Everyone should go get tested.” Medical racism is a system of oppression that works to keep Black women marginalized and perpetually in poor health. Black feminist thought would call for Black women to mobilize and push back against the oppressive system of healthcare. Collins [9] reminds us that Black women have been at the forefront of movements pushing for Black rights, women’s rights, and those for Black women. The greater the number of Black women who become aware of this systemic travesty, the more they will be empowered to fight disparities via working in healthcare, being informed consumers of healthcare, and calling out bigoted practices and practitioners.

In summary, the four themes highlighted Black women’s reactions to present and historical instances of medical racism. Our participants expressed anxiety and despair from learning more about atrocities Black women have, and continue to, face as well as revisiting stories of personal and vicarious racism. They also indicated that they felt the need to take action to improve their health outcomes despite the negative experiences that some women face(d).

## Discussion

Overall, the findings showed that knowledge of socio-cultural and historical issues and events invokes healthcare-related anxiety for Black women. This corroborates previous findings showing that Black women experience generational trauma that stems from distrust in the healthcare system [18]. And that the perception of discrimination as well as experiences of racial discrimination impacts both the physical and mental health of Black women [10, 32]. The first theme that emerged from our work was compromised mental health, whereby knowledge of historical instances of medical racism created distress among Black women. This shows that racism can impact Black women’s well-being whether it is historical, vicarious, or personally experienced. Previous research shows that Black women are exposed to implicit bias and perceived racism from healthcare professionals which impacts their interactions with providers [33]. Here, respondents noted that the historical instances of racism were a weight that they still carry as they looked out for themselves and their health. The feelings of distress brought on by reflecting on the history of medical racism provide further support for racism as a determinant of health.

The wealth and health paradox theme is supported by the literature as well. While some studies show that higher income and education levels are associated with better healthcare access, others indicate that in the case of Black Americans, this is not co. In some instances, Black women from higher SES and education levels experience higher odds of cardiovascular issues postpartum compared to low-income White women [34, 35]. Zahid et al. [34] reviewed studies from 2004 to 2019 and found White women of higher income status had lower odds of in-hospital mortality but Black women from higher income groups did not have lower odds of mortality. Findings related to the unequal incomes across similar income levels raise the concern that access to healthcare alone is not sufficient to protect Black women. Black Feminist scholarship calls for centering the needs of Black women and considering factors outside of income that may impact their lived experiences. It is clear here that race-neutral theories like the fundamental cause’s perspective, which posits that social class is the greatest predictor of health, miss nuances like racism, gendered racism, and other intersectional identities.

The role of Black women theme highlighted Black women’s awareness of their history as medical guinea pigs, willingness to take agency of their own health, and preparedness to advocate for themselves. These findings are identifiable using a Black feminist approach as Collins [9] argued that Black women’s experiences must be studied with consideration for historical context and that Black women have a passion for social justice as members of two historically marginalized groups. Additionally, the medical distrust and racism theme is prevalent in the literature, and Black women are understanding the role that they must play in advocating for improved sexual healthcare. For example, Townes et al. [36] found that Black women ages 18–35 expressed a desire to have racial and gender concordance in their healthcare provider. The study also found that Black women reported experiencing anxiety about sexual healthcare appointments due to feeling judged or being treated poorly by healthcare providers. Other researchers have reported similar findings [e.g., 1, 10, 37]. Thus, it explains why the response to perceived medical racism is connected to advocacy and women taking a role themselves in finding providers who would center their needs.

Taken together, these findings call for an expansion of healthcare to a more comprehensive care that considers the socio-cultural contexts that Black women live in, as well as their expectations for healthcare providers. The apparent fear of being profiled or stereotyped, and having knowledge of past medical-related trauma, can create barriers to accessing timely healthcare and selection of healthcare providers. A conceptual map with recommendations for healthcare providers is below. Key takeaways include recognizing the historical context of trauma, understanding that income and education are not the sole indicators of health outcomes, and building trust through transparency and cultural humility, as well as supporting advocacy efforts and respecting autonomy in health decision-making. Recognizing the historical context of trauma is vital in building connections and minimizing medical distrust among Black women. It helps in patient-provider interactions and patients feel seen and providers can acknowledge their positionality in the complex system of healthcare, and it enlightens Black women who may experience biased care with no knowledge of the root causes of those biases. Recognizing that socioeconomic status is not the sole indicator of health allows for a more intersectional understanding of patient-provider interactions and creates space to examine the role of racism as a social determinant of that. Building trust through cultural humility means looking inward. This calls for healthcare workers to ask, why do Black women fail to receive quality care on a consistent basis and what changes can we make to end that cycle? Finally, respecting autonomy in health-related decision-making means partnering with Black women patients. This means offering them several options and allowing them to make informed decisions about their own healthcare.
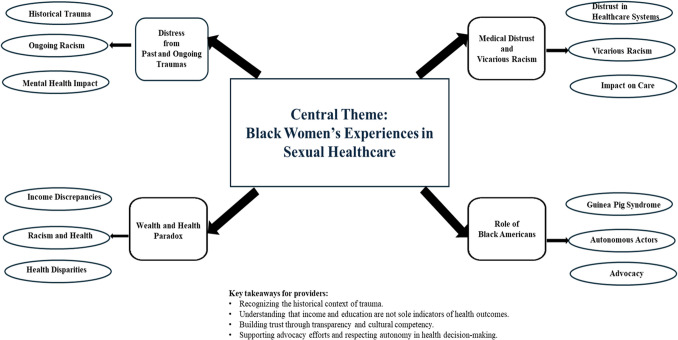


Conceptual map of medical racism as a social determinant of Black women’s sexual healthcare.

### Study Limitations

The study relied on focus group data to center the experiences of Black college women. There are several advantages to using focus group data that allows participants to build upon the responses of others; focus groups also carry disadvantages [38]. The participants could have been impacted by hearing the perspectives of other women and this biased the responses. The data analysis process is more tedious than individual interviews and requires well-trained facilitators and analysis. In our case, we took care to have a multi-investigator team, to minimize some of these limitations. The study was also limited by region and academic settings and may not reflect Black women’s perspectives from a representative community sample. We also limited the participation to heterosexual women; thus, the findings cannot be generalized to non-heterosexual women. Although we recruited broadly over social media and campus emails, only the participants who were willing to discuss the issues participated. The strength of the study, in part, lies in the research team and the Black feminist approach where we used Black women to speak to Black women about their experiences. The advisory group and research teams also were able to develop the questions together and provide a nuanced understanding of the issues and interpretations in the study.

### Future Directions

The findings of the study are limited to perception from a focus group of 28 women. Therefore, additional mixed method methodologies should be used to further examine how awareness of past and current instances of medical racism impacts Black women’s actions related to their healthcare. It is also important to engage the broader community in basic and applied research to help Black women process medical racism. Women from all socioeconomic levels should be engaged in future studies to better understand how income and education interact with the knowledge of medical racism and subsequent engagement with healthcare providers. We also recommend that medical and related health professions training must explicitly address and involve solution seeking around medical distrust and ongoing racism. Engaging with human services and mental health providers is also important to provide intervention for processing current and past traumas evoked by knowledge and experiences with medical racism. More research is needed to understand how trauma-informed interventions could support Black women’s pursuit of good healthcare.

## Conclusion

Here, we analyzed data related to Black female college student’s perspectives on present and past instances of medical racism. The themes revealed that Black women experienced distress in learning about medical racism and that these experiences, though disheartening, often propelled them to advocate for better healthcare for themselves. We conclude that the awareness of the disparities related to Black women’s sexual health outcomes and the atrocities experienced can motivate Black women to be autonomous actors in advocating for improved healthcare experiences. In other cases, Black women experience anxiety which can become a serious barrier to care. Thus, these findings are also a call to action for healthcare training and medical services that integrate cultural humility and can meet the needs of Black women.

## Data Availability

Data is not publicly available due to the sensitivity of the subject area.

## Appendix 1

**Table:**

Braun and Clark (2006) 15-point checklist of criteria for good thematic analysis		
	Braun and Clark	Current study
Transcription	The data have been transcribed to an appropriate level of detail, and the transcripts have been checked against the tapes for “accuracy”	Data was transcribed by Rev.com then checked for accuracy by research assistants
Coding	Each data item has been given equal attention in the coding process	Each response to questions analyzed here was considered and subsequently coded
	Themes have not been generated from a few vivid examples (an anecdotal approach), but instead, the coding process has been thorough, inclusive, and comprehensive	Each response to questions analyzed here was considered and subsequently coded
	All relevant extracts for each theme have been collated	Themes were created individually then collated as a team
	Themes have been checked against each other and back to the original data set	Themes were cross checked to ensure they are mutually exclusive and match the dataset
	Themes are internally coherent, consistent, and distinctive	Themes were cross checked to ensure they are mutually exclusive and match the dataset
Analysis	Data have been analyzed/interpreted, made sense of/rather than just paraphrased or described	Data have been analyzed and interpreted using a Black feminist perspective
	Analysis and data match each other/the extracts illustrate the analytic claims	Data have been analyzed and interpreted using a Black feminist perspective
	Analysis tells a convincing and well-organized story about the data and topic	Analysis conveys the impact of knowledge of historical medical racism on Black women today
	A good balance between analytic narrative and illustrative extracts is provided	The analytic narrative contains a blend of quotes, interpretations, and context
Overall	Enough time has been allocated to complete all phases of the analysis adequately, without rushing a phase or giving it a once-over-lightly	The team met weekly to discuss each step in thematic analysis, no stage was rushed
Written report	The assumptions about, and specific approach to, thematic analysis are clearly explicated	The manuscript has a brief description in text and the full checklist included as an appendix
	There is a good fit between what you claim you do, and what you show you have done/i.e., described method and reported analysis are consistent	The analysis used B&C thematic analysis as described in text
	The language and concepts used in the report are consistent with the epistemological position of the analysis	The language and concepts are all aligned with Black feminist theory as conceptualized by PH Collins
	The researcher is positioned as active in the research process; themes do not just emerge	The team of researchers actively engaged the research process and theme creation
